# Japanese nurse practitioners’ legal liability ambiguity regarding their medical practice: a qualitative study

**DOI:** 10.1186/s12912-020-00458-2

**Published:** 2020-07-09

**Authors:** Shoko Sugiyama, Kyoko Asakura, Nozomu Takada

**Affiliations:** Tohoku University, Graduate School of Medicine, 2-1, Seiryo-machi, Aoba-ku, Sendai, Miyagi 980-8575 Japan

**Keywords:** Japanese nurse practitioner, Medical practice, Nurse practitioner, Legal liability, Qualitative study

## Abstract

**Background:**

Nurse practitioners’ role is always expanding. The Japanese Nurse Practitioner (JNP) system was initiated in 2015 to shift some aspects of doctors’ work to various other healthcare professionals, including nurses. JNPs’ fulfillment of their roles was shown to have a certain degree of efficacy and provide positive outcomes for patients (e.g., shortening hospitalization period). Nurse practitioners are considered legally liable for their medical practices because they are performed on doctors’ behalf; however, in real life, there is ambiguity regarding such practice. It is necessary to clarify nurse practitioners’ legal liability in order to ensure the safety of their medical practice and protect them in medical procedures performed on physicians’ behalf. This study aimed to clarify how JNPs understand their own legal liability in medical practice.

**Methods:**

A qualitative, inductive research design was adopted to record participants’ opinions. The survey was conducted from October 2017 to February 2018. Participants were nurses working as JNPs at general hospitals in eastern Japan. We recruited participants via snowball sampling.

**Results:**

With regard to JNPs’ legal liability in their medical practice, three themes understanding were observed: “determining whether the JNP has the ability to perform the assigned medical procedure,” “exercising caution when performing medical procedures on a doctor’s behalf” and “an urge to follow up with appropriate medical practice until the end of care.”

**Conclusions:**

We demonstrated that JNPs recognized their own legal liability in medical practice. They had to protect themselves because their legal position was ambiguous. Furthermore, JNPs accepted that diagnosis and drug prescription could be performed on behalf of doctors if trusting relationships had been previously established.

## Background

Nurse practitioners’ (NPs) role is always expanding. The scope of the medical practice performed by NPs varies somewhat from country to country and state to state, and NPs are able to perform certain levels of diagnosis and treatment autonomously [1]. Moreover, they compensate for doctor shortage and provide medical care in areas where such shortage exists [2]. For example, although there are some differences between countries, NPs in the United States [3], the United Kingdom [4], Canada [5], and Australia [6] are permitted to perform diagnoses, order tests, and prescribe medications. The Japanese Nurse Practitioner (JNP) system was initiated in 2015 to shift some aspects of doctors’ work to various other healthcare professionals, including nurses [7].

Depending on how JNPs fulfill the requirements of a role, effectiveness measurements, such as shortening the hospitalization period for patients and early response to emergencies [8, 9], have been observed for patients and institutional users: For example, in cases where NPs care for adult patients with chronic renal failure, the effective management of blood pressure, cholesterol, and blood sugar levels was found to be outstanding compared to general care [10]. Additionally, in nursing homes, NP intervention was shown to significantly reduce the number of times emergency transportation was used and to shorten the duration of hospitalization for patients [11]. Moreover, when the actions of NPs and physicians were compared in emergency rooms, there was no significant difference found in the number of tests ordered in triage, and the small difference observed did not affect patients’ overall time spent in emergency room beds [12]. NPs possess a high degree of knowledge [5], are able to make quick diagnoses and judgments [6], can respond to changes in patients’ conditions [7, 10, 11], and provide patients with necessary medical care on behalf of doctors [2–7, 10, 11].

Moreover, NPs are considered legally liable for their medical practices because they are performed on doctors’ behalf; nonetheless, in reality, there is some ambiguity regarding the exact situations in which NPs can be held liable for their medical practice. For example, in cases in which NPs and physicians work together, although NPs’ activities are evaluated by the doctor, there is some overlap in their scope of practice (i.e., NPs can prescribe drugs and perform some, but not all, advanced medical practices) [13], making the issue of legal liability vague [14]. Additionally, NPs are legally unable to independently perform advanced medical procedures [15]. Although NPs are an integral part of medical teams in emergency departments, they must have sufficient practical ability to ensure the medical practice safety and avoid litigation [16]. Nonetheless, given the fact that NPs hold a large amount of responsibility regarding their practice and that the number of patients to whom an NP responds increases in a daily basis, the number of medical malpractice insurance claims has also been increasing in countries such as the United States, where such litigation is particularly commonplace [17]. Hence, it is necessary to clarify NPs’ legal liability so as to ensure the safety of medical procedures and protect NPs regarding their medical practices performed on physicians’ behalf.

In Japan, discussions on the JNP system are ongoing. Currently, JNP refers to a nurse who has undergone specific training in a master’s program, has a master’s degree, and is certified by Japanese Organization Nurse Practitioner Faculties. JNPs may be more responsible for performing medical procedures than a non-trained nurse. Although the Public Health Nurses, Midwives, and Nurses Law was partially revised due to the enforcement of the JNP system in 2015, doctor instruction remained necessary when JNPs provided medical treatment even after the law’s revision. Despite their specialized training, JNPs cannot make judgments regarding the practice of medical treatments on their own, and, like general nurses, assist in medical action under the direction of a doctor. It can be construed that JNPs may be more liable than a general nurse if a medical accident results from their medical assistance. According to the Public Health Nurses, Midwives, and Nurses Law, when a medical accident occurs due to an action performed by JNP under the direction of a doctor, both the doctor and JNP can be interpreted as legally responsible. This increased liability may result from JNPs having specific training, a master’s degree, and more advanced knowledge and skills. As a result, under the current JNP system, JNPs cannot assess a patient’s condition and decide on the necessary medical action, but face a situation where legal liability is found for medical accidents performed under a doctor’s instruction.

At this stage, JNPs’ legal responsibility regarding medical actions is ambiguous. JNPs are not self-employed. Because they are hired by a hospital, their practices, and thus the scope of their liability, vary according to the hospital’s regulations. To date, there have been no cases where JNPs have been held liable in Japan. However, if a regular nurse is involved in a medical accident, the nurse, the doctor who issued the instructions, and the hospital that employs the nurse are legally liable. Similarly, if a problem arises concerning the actions taken by a JNP, the legal responsibility may be imposed on the JNP, the doctor who issued the instructions, and the employer’s hospital [18]. Thus, this study aimed to determine how JNPs understand their own legal liability in medical practice. The results provide suggestions regarding patient safety and NPs’ legal protection. Additionally, knowledge accumulation on the topic can help expand the role of JNPs in the medical setting.

## Methods

### Research design

Nursing practice is a fluid phenomenon that is influenced by various contexts, such as the human influences of nurses, patients, or medical professionals; environmental issues; and the situations surrounding the practice itself. However, it is difficult to control so many contexts in research. Therefore, we used a qualitative and inductive research design that could comprehensively capture the phenomena of JNPs’ thought processes and the surrounding context. This design allows for better understanding the wholeness of a phenomenon and for discovering its inherent depth, richness, and complexity via its meaning [19]. The study was also conducted as a preliminary survey to examine nurses’ responsibilities. This study was conducted using researchers and a PhD candidate with experience in qualitative data collection and analysis. These researchers were continuously involved in the research planning, data collection, data analysis, and manuscript writing phases of the study.

### Setting and participants

The survey was conducted from October 2017 to February 2018. Participants were three nurses working as JNPs at general hospitals in the eastern region of Japan. Researchers explained the purpose of the study to six JNPs and invited them to participate in the study, and three consented to do so. These three JNPs belonged to the cardiac surgery, respiratory medicine, and emergency departments. Each JNP had between 9 and 22 years of experience as nurses in addition to 2–5 years’ experience as an NP. However, in certain hospitals, even if one has obtained the JNP qualification, situations in which working as a JNP at one’s affiliated hospital is not permitted could arise. The inclusion criterion for participation in the study was being an active JNP employee for more than 2 years. We recruited participants via snowball sampling.

### Data collection

Data were collected via participant observation and interviews. Researchers initially observed JNPs and took field notes on how JNPs practice nursing. We did so because nurses are not always aware of their legal liability during their own practice and because it would be easier to reconstruct specific and factual scenarios during subsequent interviews. The observed scenarios included handovers between JNPs, confirmation of a doctor’s instruction, conferences with medical staff, and patient-related practices. Observations were conducted for 2–3 days for each JNP. Individual interviews were conducted once (or twice, if a follow-up was warranted) for each JNP and took place in rooms in which privacy could be maintained. Each interview lasted approximately 1 h. Based on the field notes, we asked JNPs to think about their own legal liability, and we used certain examples of their medical practice acquired through observation. Each interview was recorded with the participant’s permission.

### Data analysis

We comprehensively described JPNs’ legal liability with regard to certain medical procedures. The theme of the analysis was “how JNPs understand their own legal liability in medical practice.” We minimized bias as much as possible as we proceeded with our research. In particular, when analyzing data, it is possible that researcher bias could arise in data interpretation; therefore, after creating specific conclusions about the data, we analyzed their appropriateness by constantly returning to the actual data. Further, to minimize such bias, we were supervised by researchers with experience in qualitative research and nurses with experience in nursing research. This was done to ensure the credibility and validity of the study content and results.

## Results

### JNPs’ understanding of legal liability in medical practice

The results of the analysis of JNPs’ legal liability in their medical practice revealed three themes: “determining whether the JNP has the ability to perform the assigned medical procedure,” “exercising caution when performing medical procedures on a doctor’s behalf,” and “an urge to follow up with appropriate medical practice until the end of care.”

“Determining whether the JNP has the ability to perform the assigned medical procedure”

With regard to the first theme of understanding, data showed that even if a JNP was requested or permitted by a doctor to perform a medical procedure on that doctor’s behalf, the JNP was responsible for assessing the patient’s condition, identifying procedure should be performed and how, and determining whether to perform the medical procedure. Depending on the type of medical procedure involved, even if there was no problem with a JNP’s ability to perform the procedure, it was possible that the patient’s state could suddenly change to one that would require a doctor because of procedure implementation. Therefore, even if JNPs were allowed to perform a certain medical procedure by a doctor, they might not do so if they judged it to be dangerous.

*In medical practice, it cannot be said that accidents will definitely not occur. Specifically, when performing invasive procedures in place of the doctor, the doctor says, “I leave everything to you, so you can do anything.” However, if any complications occur after medical procedures, the doctor may say, “You were the one who did this.” In that case, it becomes difficult, because I’m forced to accept legal liability. When it comes to legal liability, I feel that I have to be careful when dealing with it.* (NP2)

*I only choose to perform medical procedures that do not entail complications, so I haven’t performed a peripheral insertion of a central catheter, I never perform such difficult medical procedures. In other words, I restrict myself. There are 38 medical procedures I am qualified to perform, but I do not do all of them. I will not even do the exchanges required for gastric fistulas. I try to simulate the procedure in my head and do hands-on medical practice. Once I become confident, I can carry out the medical procedure while being occasionally observed by a doctor. I think that I cannot perform medical procedures that have a high risk, and I do not perform medical procedures that I deem risky.* (NP3)

“Exercising caution when performing medical procedures on a doctor’s behalf”

For the second theme, the data showed that when JNPs were tasked with performing medical procedures on behalf of a doctor that were not among their specified 38 performable medical tasks, they experienced anxiety about whether or not they should be performing the tasks and felt pressure to not make mistakes while doing so. By law, JNPs do not have the authority to directly provide a diagnosis and/or prescription. However, when doctors had JNPs act as their substitutes, the latter provided patients with diagnoses and prescriptions. Although these actions performed on behalf of a doctor were ultimately confirmed by the doctor, who remained responsible for determining the content of the orders, the JNPs were required to input their own ID numbers to implement these medical procedures (i.e., diagnosis and prescription). This requirement led to JNPs feeling pressured to not make mistakes, mainly due to the anxiety evoked by the possibility of being legally liable if mistakes occurred.

*For cases such as fractures, I write down the diagnoses. I have to write things like “there is a fracture here” on behalf of a doctor, so I cannot afford to make any mistakes. That is my number one [priority]. Because there cannot be any mistakes, I feel that I have to write diagnoses down properly. I cannot write down any misdiagnoses.* (NP 3)

*I prescribe medicine on my own, so it is very scary. I cannot prescribe medicines that I am unfamiliar with, so, in such cases, I ask somebody—for example, pharmacists or doctors who are familiar with the medicine. When my doctor tells me to prescribe medicine, I ask the doctor why this medicine is necessary … Because I am the one giving instructions for the medicine; it is scary to prescribe it without knowing the patient’s state or the effect of the drugs.* (NP 1)

“An urge to follow up with appropriate medical practice until the end of care”

For the third theme of liability understanding, the data showed that JNPs were concerned about patients’ progress after they had performed medical practices on them, and they wanted to ensure that such patients were followed up with until the end of their hospital stay, so as to not leave anything unfinished. Although JNPs cannot directly treat their patients, their responsibility was signified by repeatedly confirming the progress of the patients involved in their medical practices. Furthermore, in understanding the changes that resulted from their direct actions on patients, JNPs were able to more clearly comprehend their legal liability.

*Basically, I see my patients the whole time until they are discharged. Occasionally, I go to see patients when they come in for outpatient care. I ask them “How are you doing? Are you taking care of yourself?” Sometimes the patients come to see me. I am involved in [protecting] the lives of the patients, so I am worried about them … I think that for the patients, hospitalization is a turning point in their lives. It is a life-changing experience, so I wonder how patients’ lives have changed and how they are doing.* (NP1)

*I think that it is ideal to check and assess bed sores and debridement every day, but I cannot follow up on this, because I can only check and assess those conditions once a week. I have to leave it to the doctors or other nurses, but I hesitate leaving those tasks to others … The patient’s condition may be alright when I assess them, but infection, bleeding, or something else could set in later on. These things are real possibilities, so I think I need to be somewhat cautious. If our hospital was not so far from the patients, we would be able to respond to them more quickly.* (NP2)

## Discussion

### Ambiguity of JNPs’ position in the legal system

Our results indicated that JNPs carefully determined whether they were able to perform certain medical procedures and assessed the difficulty of a medical practice when they were asked to perform tasks on a doctor’s behalf. Our results were identical to those of a previous study showing that the law was a barrier to NPs’ independent practice [15]. JNPs practice medical procedures in training; therefore, they are able to implement the procedures if they intend to do so [20]. However, if complications occur owing to the performed medical procedures, additional medical intervention is required to treat the complications. The medical procedures that NPs perform are carried out under a doctor’s directions. However, any additional medical care was regarded as going beyond the range of actions that can be performed by the JNPs’ own judgments or by them under a physician’s direction. Moreover, JNPs reportedly considered whether the medical procedures they performed caused complications, and whether they needed to perform further medical procedures to minimize patient’s risks.

Based on our observations, we believe that JNPs’ follow up of the medical procedures they have performed was an expression of their perceived responsibility. In team-based medical care, where cooperation exists between doctors and pharmacists, it becomes difficult to clarify JNPs’ scope of responsibility and roles when performing specific procedures [21]. Even in Japan, the existence of JNPs in medical practice is not widely implemented, and it has been shown that ascertaining JNPs’ role is a difficult task [22]. In the current study, the range of medical procedures performed by JNPs differed between facilities and medical departments. With respect to the unclear scope of JNPs’ role, this may be owed to the fact that each facility or department recognizes the scope of activities that this professional group should perform in different ways; namely, there is inconsistency between these institutions. In situations where JNPs’ role is unclear, it may be assumed that societal reaction to the failure of a medical procedure would differ based on who performed the actions that led to the failure—a doctor or a JNP (despite the fact that, to date, there have been no cases in which a JNP who caused a medical accident was subject to a lawsuit). Given the fact that Japanese law recognizes and allows for physicians to perform medical procedures, this also implies that they are liable for the procedures they perform. Contrastingly, JNPs’ role is not clearly recognized by Japanese law, despite this professional group’s desire for this legal liability to be more clearly defined. Therefore, the issue surrounding responsibility for medical procedures performed by JNPs under the instruction of physicians requires further consideration with respect to who should be held legally responsible for medical accidents, suggesting the need to further explore individual practitioners’ authority and scope of obligations.

### Relationship between JNPs and physicians

Results showed that JNPs felt pressured when performing medical procedures on doctor’s behalf. Particularly, JNPs felt pressured to diagnose and prescribe medication to patients on physician’s behalf. It may be assumed that JNPs accepted performing these activities on doctor’s behalf because they wished to fulfill the latter’s expectations, and the pressure to avoid misdiagnoses or incorrect prescriptions may have come from their intent to not bother their superiors (doctors). Further, we believe that, in our sample, doctors requested for JNPs to perform such activities in consideration to their abilities and because they are subordinates. Finally, we think that although the roles of JNPs are somewhat ambiguous with respect to diagnoses and prescriptions, they manage to handle the pressure of being a substitute for doctors by establishing trusting relationships with them.

When a doctor instructs a JNP to prescribe a drug that is unknown to the JNP, the JNP typically checks the drug information, such as its efficacy for the patient’s symptoms, side effects, and proper use, with a doctor or pharmacist. A previous study has shown that comprehensive drug evaluations by NPs promote the effective symptom management of patients and effective medical economy regarding drug prescription tailored to patients’ needs [23]. Including such tasks within the educational and training curricula of JNPs, whereby they can modify the dosage of drugs or change the drug to be administered according to the patient’s condition, may lead to enhanced treatment and nursing tailored to each patient’s condition.

### Limitations of the study

The results of this study are based on interviews and observations of three JPNs working in hospitals; it was difficult to recruit research participants because in Japanese hospitals, JNPs often work under doctors’ supervision, and the doctors tend to not grant permission for participation. Moreover, JNPs have not had the opportunity to demonstrate their understanding of all legal responsibilities, as no lawsuits have yet been filed. However, these findings provide a better understanding of JNP’s roles and actual working conditions and suggest that their responsibilities need to be further clarified.

## Conclusions

Through this study, we clarified that the JNPs in our study recognize their own legal liability in medical practice; their legal responsibility regarding the implementation of medical procedures was based on three recurring themes that appeared in the qualitative analyses we performed: 1) “determining whether the JNP has the ability to perform the assigned medical procedure,” 2)“exercising caution when performing medical procedures on a doctor’s behalf,” and 3) “an urge to follow up with appropriate medical practice until the end of care”.

Our results showed that JNPs were in a situation in which they had to protect themselves because their legal position was vague. Further, even if they performed legally ambiguous medical procedures, they still chose to fulfill their responsibility as JNPs and performed these procedures. We surmise that if JNPs’ legal liability scope was more clearly defined or if JNPs’ roles were to be expanded, perhaps they would be able to fulfill their tasks without feeling anxious as to whether or not they are operating within the scope of their legal liability.

## Data Availability

Datasets generated and analyzed during the current study are not publicly available but can be made available from the corresponding author upon reasonable request.

## Abbreviations

JNP: Japanese nurse practitioner
NP: Nurse practitioner
